# System Integration and Participant-Level Validation of a Gate-Based Contactless Multimodal System for Railway Pre-Duty Screening

**DOI:** 10.3390/s26185805

**Published:** 2026-09-13

**Authors:** Cheolwoo Lee, Taemyoung Yin

**Affiliations:** 1 SAFETYLAB Co., Ltd., 18 Bucheon-ro 198beon-gil, Wonmi-gu, Bucheon-si 14547, Republic of Korea; headgdo@naver.com; 2 Department of Railway Driving Systems Engineering, Songwon University, Gwangju 61756, Republic of Korea

**Keywords:** railway safety, pre-duty screening, contactless physiological monitoring, remote photoplethysmography, multimodal sensing, blood pressure estimation, breath alcohol detection, occupational health screening, Bland–Altman analysis

## Abstract

**Highlights:**

**What are the main findings?**

- A gate-based Through-Pass system integrated identity verification, multimodal contactless sensing, signal-quality control, and rule-based screening/referral, with sensor acquisition completed over approximately 15 s per attempt.
- In 300 participants, the evaluated measurements showed mean biases close to zero and high concordance with reference measurements; no consistent associations of error with age, sex, body mass index, grouped Fitzpatrick skin type, or eyeglass use were detected.

**What are the implications of the main findings?**

- The integrated platform supports further evaluation of contactless multimodal measurement for railway pre-duty assessment without requiring wearable sensors during acquisition.
- Field validation under operational railway conditions, independent external validation of the blood pressure estimator, and validation of the screening classifications are required before routine implementation.

**Abstract:**

Railway pre-duty assessments require rapid, objective measurements that do not disrupt operational workflows. This study evaluated the system-level integration and participant-level measurement performance of a gate-based Through-Pass platform for railway pre-duty screening. The platform combines radio-frequency identification, infrared temperature sensing, camera-based remote photoplethysmography, breath-alcohol sensing, signal-quality control, edge processing, and rule-based screening. Participant-level method comparison was performed in 300 adults using paired Through-Pass and reference measurements of body temperature, heart rate, systolic and diastolic blood pressure, and blood alcohol concentration. Agreement was assessed using Bland–Altman analysis, concordance statistics, error metrics, Deming regression, and multivariable models of measurement error. Mean biases were close to zero for all five outcomes, and Lin’s concordance correlation coefficients ranged from 0.938 to 0.995. After multiple-testing correction, measurement error showed no consistent associations with age, sex, body mass index, grouped Fitzpatrick skin type, or eyeglass use, whereas the reference-value magnitude was associated with error for diastolic blood pressure and blood alcohol concentration. These findings support the feasibility of integrating established sensing components into a unified pre-duty measurement platform under controlled indoor conditions; field and screening-classification validation remain necessary before operational implementation.

## 1. Introduction

Railway transportation is a safety-critical system in which operational failures can affect passengers, employees, and the surrounding communities. Although advances in infrastructure, signaling, rolling stock, and automated control have reduced many equipment-related risks, human performance remains an important contributor to railway accidents and operational incidents [1,2,3]. Acute conditions such as fatigue, insufficient sleep, and alcohol consumption may impair vigilance, reaction time, situational awareness, and decision-making, thereby increasing the likelihood of operational error [4,5]. Assessing an operator’s condition immediately before duty is therefore an important component of preventive railway safety management.

In the Republic of Korea, railway workers engaged in driving, traffic control, and other designated safety-critical duties are required to undergo periodic physical and aptitude examinations, and specified railway workers are prohibited from performing their duties under the influence of alcohol or drugs [6]. A three-round Delphi study involving 20 experienced Korean railway-driving professionals identified 14 factors relevant to pre-duty work-suitability assessment, including alcohol use, fatigue, medication use, illness, sleep, stress, vision, hearing, and physiological condition [7]. Alcohol use can be assessed objectively through pre-duty breath testing [8], whereas fatigue- and sleep-related fitness are commonly evaluated using subjective self-report measures [9]. Such self-assessment may fail to identify reduced functional capacity when workers underestimate fatigue, are unaware of a physiological abnormality, or are reluctant to report a condition that could delay duty assignment [4,9]. These limitations support the use of rapid objective measurements as an adjunct to, rather than a replacement for, broader professional assessment of work suitability.

Contactless physiological sensing offers a potential means of obtaining objective measurements without substantially disrupting operational workflow. Remote photoplethysmography (rPPG) derives cardiovascular information from subtle pulse-related changes in light reflected from the skin and recorded using a camera [10,11,12]. Chrominance-based signal processing, motion assessment, and related analytical methods have improved camera-based heart-rate estimation under varying acquisition conditions [13,14]. Camera-derived signals have also been investigated for estimating blood pressure and other physiological parameters relevant to health screening [15,16]. Because rPPG does not require electrodes, cuffs, finger probes, or wearable devices during signal acquisition, it may be particularly useful in settings where many individuals must undergo brief physiological assessments.

Physiological monitoring has already been investigated in transportation and other safety-critical occupations. Wearable electroencephalography has been used to evaluate vigilance during high-speed railway operation [17], and wearable systems have been studied for fatigue monitoring in mining and other industrial environments [18,19]. Contactless approaches have also advanced beyond healthcare applications. Tsai et al. developed a vision-based rPPG system for noncontact assessment of driver fatigue [20], while Guo et al. evaluated an in-vehicle contactless vital-sign monitoring system using near-infrared time-of-flight imaging under highway and local-road conditions, explicitly addressing motion and changing ambient illumination [21]. More recently, multimodal camera-based approaches have combined rPPG-derived physiological information with facial and motion features for non-invasive driver-fatigue assessment [22].

These transportation-oriented systems demonstrate the feasibility of contactless physiological monitoring in operational settings but primarily focus on continuous or repeated assessment during vehicle operation. The Through-Pass platform addresses a complementary stage of the safety workflow: brief, stationary assessment before duty begins. Its intended contribution is therefore not a new individual sensing modality or fundamental signal-processing algorithm, but rather the system-level integration of participant identification, multimodal contactless sensing, automated signal-quality assessment and reacquisition, edge processing, data communication, and rule-based screening/referral within a single gate-based workflow. This distinction is important because the requirements for pre-duty screening differ from those for continuous driver monitoring, particularly with respect to rapid acquisition, identity linkage, and the handling of measurements that do not satisfy predefined quality criteria.

Evaluation of such an integrated platform requires more than demonstrating a correlation between a system measurement and a reference instrument. Measurements must be linked to the correct participant, unsuitable recordings should be identified before physiological estimates are reported, and method comparison should quantify both systematic bias and individual-level agreement. Bland–Altman analysis is specifically designed to evaluate differences between measurement methods and is therefore more informative for this purpose than correlation alone [23]. Camera-derived physiological measurements may also be influenced by motion, illumination, facial occlusion, skin reflectance, and measurement magnitude [13,14,24]. These factors are particularly relevant when physiological measurements may subsequently contribute to threshold-based screening decisions.

In this study, we evaluated the system-level integration and participant-level measurement performance of a gate-based Through-Pass platform combining radio-frequency identification, infrared temperature sensing, camera-based rPPG, blood pressure estimation, contactless breath-alcohol sensing, edge processing, signal-quality control, and rule-based screening/referral. Multimodal sensor acquisition was performed over approximately 15 s per attempt, with reacquisition triggered when predefined quality criteria were not met. Paired Through-Pass and reference measurements of body temperature, heart rate, systolic and diastolic blood pressure, and blood alcohol concentration were evaluated in 300 adults using Bland–Altman analysis, concordance statistics, Deming regression, and participant-level error models. The present study was designed to evaluate measurement agreement under controlled indoor conditions; it did not evaluate robustness under operational railway-site disturbances or validate the FIT, CONDITIONAL, and UNFIT classifications against independent duty-fitness or safety outcomes. These latter aspects require dedicated field and classification validation before routine operational implementation.

## 2. Materials and Methods

### 2.1. Study Design

This study comprised system development followed by participant-level method comparison. First, a Through-Pass multimodal screening system was developed to integrate identity verification, contactless physiological sensing, signal-quality control, edge processing, data communication, and rule-based screening within a single gate-based workflow. Second, agreement between measurements obtained using the Through-Pass system and corresponding reference instruments was evaluated in an expanded cohort of 300 adults. The evaluation dataset included paired measurements of body temperature, heart rate, systolic blood pressure, diastolic blood pressure, and blood alcohol concentration. Recorded participant characteristics included sex, age, height, weight, body mass index (BMI), Fitzpatrick skin type, and eyeglass use.

For blood pressure, measurements collected within the broader data-collection program contributed to population-level model calibration and refinement. Accordingly, the blood-pressure comparison in the present study represents an internal participant-level performance evaluation rather than a fully independent external validation. Peripheral oxygen saturation (SpO_2_) measurement capability was implemented in the prototype; however, paired reference SpO_2_ measurements were not collected in the present study. SpO_2_ was therefore excluded from the quantitative agreement analyses, and the present study does not evaluate or claim the accuracy of this functionality.

### 2.2. Through-Pass Screening System

**Figure 1:**
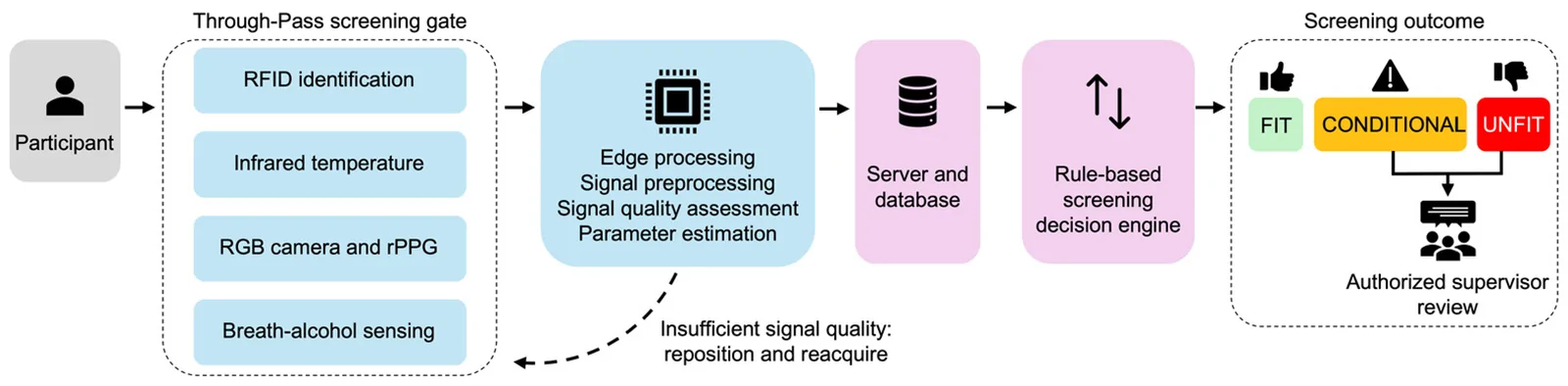
Overall architecture and operational workflow of the Through-Pass multimodal pre-duty screening system. A participant enters the screening gate, where RFID, infrared temperature measurement, facial video acquisition for rPPG, and breath-alcohol screening are performed. Edge processing conducts signal preprocessing, signal-quality assessment, and physiological parameter estimation. Accepted measurements are transmitted to a server and database and evaluated by a rule-based screening decision engine. The system displays FIT, CONDITIONAL, or UNFIT outcomes, with CONDITIONAL and UNFIT outcomes referred for authorized supervisor review. Insufficient signal quality triggers participant repositioning and reacquisition. Arrows indicate the direction of the screening workflow and data transfer.

The Through-Pass system was designed as a gate-based platform for identity verification and multimodal pre-duty screening, with sensor acquisition completed over approximately 15 s per attempt. Accordingly, the 15 s acquisition period represents the duration of a single measurement attempt and not the total screening time when reacquisition is required. The prototype integrated a CR100 RFID reader (Hyunseung Korea, Seoul, Republic of Korea), an ES-R200BS infrared temperature sensor (EHOO SYS Co., Ltd., Seoul, Republic of Korea), a C920 HD Pro Webcam RGB camera (Logitech, Lausanne, Switzerland) for rPPG, an ES-H00 contactless breath-alcohol sensor (EHOO SYS Co., Ltd., Seoul, Republic of Korea), an ES-BK32 embedded processing computer and display (EHOO SYS Co., Ltd., Seoul, Republic of Korea), and long-term evolution (LTE) communication with a rule-based screening decision module (Figure 1). The screening sequence was initiated by RFID-based identification. Facial video and sensor measurements were then acquired, processed, and evaluated for signal quality. Measurements that did not satisfy the predefined quality criteria were rejected, and the participant was prompted to reposition and repeat the acquisition. Accepted measurements were transmitted to the screening decision module, and the resulting FIT, CONDITIONAL, or UNFIT classification was displayed on the user interface. CONDITIONAL and UNFIT outcomes were referred for authorized review rather than being treated as final automated decisions. The classification module was included to describe the intended screening workflow; however, the present study did not evaluate the validity of the FIT, CONDITIONAL, or UNFIT classifications against an independent duty-fitness criterion or operational safety outcomes. Classification sensitivity, specificity, and predictive performance were therefore outside the scope of the present study.

### 2.3. rPPG Acquisition and Signal Processing

Facial video was acquired over a 15 s period at 30 frames/s. Facial landmarks were detected using MediaPipe Face Mesh (Google LLC, Mountain View, CA, USA), and regions of interest (ROIs) were selected from the forehead and both cheeks. Mean red, green, and blue intensity values were calculated for each frame to generate temporal RGB signals. The RGB signals were detrended and standardized before application of the chrominance-based rPPG extraction method (CHROM). The resulting rPPG waveform was filtered using a second-order Butterworth bandpass filter from 0.7 to 3.0 Hz, corresponding to a heart-rate range of 42–180 beats/min. Heart rate was estimated from the dominant frequency identified by fast Fourier transform (FFT).

Frequency-domain signal-to-noise ratio (SNR) was calculated as a signal-quality descriptor. Operational quality control and reacquisition were based on facial ROI stability, frame stability, acquisition validity, and successful generation of an rPPG result. Facial movement was quantified as the displacement of the current ROI center from its initial position, normalized by the longer dimension of the initial facial ROI. A normalized displacement of ≤0.15 was considered stable and the corresponding RGB sample was retained. For displacement >0.15 and <0.30, ROI tracking continued, but the corresponding frame was excluded. A displacement ≥0.30 or failure to detect the face was treated as a hard-fail event; five consecutive hard-fail events caused the acquisition to be terminated. For a 15 s acquisition, frame-stability criteria required at least 360 valid samples, a valid-acquisition rate of ≥80%, and a mean effective frame rate of ≥24 frames/s. Acquisition was also considered unsuccessful if the measurement duration or frame rate was invalid or if the rPPG algorithm failed to generate a valid result. Measurements failing any required quality criterion were rejected, and the participant was prompted to reposition and repeat the acquisition. Only recordings that passed the quality-control step were used for physiological parameter estimation.

The system also calculated RGB-derived luminance as an environmental quality indicator instead of measuring illumination directly in lux. The resulting lighting-quality score incorporated mean luminance, temporal stability, left–right facial illumination uniformity, luminance drift, and the proportion of samples outside the predefined luminance range. The present study did not quantify initial-acquisition rejection rate, reacquisition frequency, or final acquisition failure rate; these operational metrics therefore remain to be evaluated prospectively during field deployment.

### 2.4. Blood Pressure Estimation Model

Systolic and diastolic blood pressures were estimated from rPPG-derived features obtained from recordings that passed the signal-quality criteria described above. The blood-pressure modeling framework evaluated multiple regression approaches, including Ridge regression, support vector regression, Random Forest, XGBoost, and LightGBM. Candidate models were assessed using prediction-error metrics including mean absolute error (MAE), root mean square error (RMSE), and coefficient of determination (R^2^). Qualifying model outputs were combined within a weighted ensemble framework, with model weights selected to reduce the MAE of the combined prediction.

Model inputs were derived from facial RGB/rPPG signals, together with physiological, environmental, and signal-quality information. Representative feature identifiers included r_mean, r_std, g_mean, g_std, b_mean, b_std, chrom_hr, chrom_snr, luma_mean, motion_shift_mean, and roi_stability_ratio. Heart rate, body temperature, illumination level, and signal-quality indicators were also available as auxiliary inputs.

Reference systolic and diastolic blood pressure values used during model development were obtained using cuff-based blood pressure measurements. Two to three repeated reference measurements were obtained for label generation, with the mean or median used as the reference label. Calibration was performed at the population level rather than through participant-specific calibration; individual users were not required to provide a personal baseline blood pressure before estimation.

Measurements collected within the broader data-collection program contributed to population-level model calibration and refinement. Accordingly, the blood-pressure comparison reported in the present study represents an internal participant-level performance evaluation rather than fully independent external validation. The exact participant-level training/validation split and development-cohort performance metrics were not available in sufficiently documented form and are therefore not reported. Independent external validation of the blood pressure estimator remains necessary.

### 2.5. Blood Alcohol Estimation

Blood alcohol concentration (BAC) was estimated using an MQ-3 tin-oxide semiconductor alcohol sensor (Zhengzhou Winsen Electronics Technology Co., Ltd., Zhengzhou, China) integrated into a contactless breath-sampling module. Sensor response was converted to an estimated ethanol concentration using calibration parameters obtained from standard ethanol-gas measurements. The MQ-3 module was intended for initial screening and was not used as an evidential breath analyzer. Dedicated cross-interference challenge testing using potentially interfering volatile compounds was not performed in the present study; therefore, this study assessed agreement between MQ-3-derived BAC estimates and the corresponding reference measurements under the study conditions rather than the analytical selectivity of the MQ-3 sensor.

### 2.6. Participants and Evaluation Protocol

The evaluation study was conducted at SAFETYLAB Co., Ltd., in Bucheon, Republic of Korea, from 25 to 29 July 2026. A total of 300 adults, including railway personnel and other adult volunteers, participated in the study. Eligible participants were at least 18 years of age and able to complete the measurement protocol. Individuals who reported an acute condition or symptoms likely to interfere with measurement on the study day, such as fever or clinically significant cardiovascular symptoms, were excluded. Eligibility was assessed by self-report, and all participants provided written informed consent before data collection. Measurements were conducted under controlled indoor conditions intended to approximate a railway crew facility. Ambient illumination was maintained at approximately 300–500 lux, room temperature at 20–24 °C, and relative humidity at 40–60%. These controlled conditions were selected to support participant-level method comparison and did not reproduce railway-site disturbances such as rapidly changing illumination, structural vibration, or uncontrolled participant movement. Participants were allowed to rest briefly under the test conditions before measurement and were positioned at the designated location within the gate, facing the RGB camera and infrared temperature sensor. The Through-Pass system acquired contactless measurements during an approximately 15 s acquisition period per attempt. Corresponding reference measurements were obtained during the same study visit and as close in time as practicable to the Through-Pass measurements. Recordings that did not satisfy the rPPG quality criteria were repeated after participant repositioning. Height and weight were self-reported, and BMI was calculated as weight in kilograms divided by height in meters squared. Fitzpatrick skin type and eyeglass use were recorded to evaluate participant-related variation in measurement error.

### 2.7. Reference Measurements

Reference measurements were obtained during the same study visit and as close as practicable to the corresponding Through-Pass measurements. Reference body temperature was measured using a calibrated FS-300 noncontact infrared thermometer (HuBDIC Co., Ltd., Anyang-si, Republic of Korea). Reference heart rate was obtained as the pulse rate measured by a BPBIO320 automated blood pressure monitor (InBody Co., Ltd., Cheonan, Republic of Korea). Reference systolic and diastolic blood pressures were measured using the same BPBIO320 device with an appropriately sized upper-arm cuff. Reference blood alcohol concentration was measured using a fuel-cell-based breath-alcohol analyzer (Sentech Korea Corp., Paju-si, Republic of Korea). All reference devices were operated according to the manufacturers’ instructions and functionally checked before use.

### 2.8. Outcome Measures

The primary outcomes between the Through-Pass system and the corresponding reference measurements for body temperature, heart rate, systolic blood pressure, diastolic blood pressure, and blood alcohol concentration were in agreement. For each participant and outcome, the signed measurement error was defined as follows:

Through-Pass measurement − reference measurement.

Absolute error was defined as the absolute value of the signed difference. Additional agreement outcomes included mean bias, 95% limits of agreement, mean absolute error, root mean square error, Lin’s concordance correlation coefficient, Pearson correlation coefficient, Deming-regression slope and intercept, and proportional bias. Secondary analyses evaluated whether absolute measurement error was associated with age, sex, BMI, grouped Fitzpatrick skin type, eyeglass use, or reference-measurement magnitude. The performance of the FIT, CONDITIONAL, and UNFIT classifications was not included as an outcome of the present study.

### 2.9. Statistical Analysis

Participant characteristics were summarized as the mean and standard deviation for continuous variables and the number and percentage for categorical variables. Agreement between the Through-Pass system and the corresponding reference instrument was evaluated separately for each measurement outcome.

Mean system and reference values, mean bias, the 95% confidence interval for the bias, 95% limits of agreement, mean absolute error, and root mean square error were calculated. Lin’s concordance correlation coefficient and its 95% confidence interval were used to quantify concordance. Pearson correlation was reported as a measure of linear association and was not interpreted as evidence of agreement. Deming regression was used to estimate the relationship between the Through-Pass and reference measurements, allowing for measurement error in both variables. The primary analyses assumed an error-variance ratio of λ = 1, corresponding to equal measurement-error variances for the two methods. Because replicate measurements sufficient to estimate method-specific error variances were not available, λ was not estimated empirically. Sensitivity analyses were therefore performed using λ = 0.5 and λ = 2 to evaluate the robustness of the Deming-regression slopes to alternative variance-ratio assumptions.

Bland–Altman plots were used as the primary graphical assessment of individual-level agreement. For each participant, the signed difference was plotted against the mean of the paired system and reference measurements. Proportional bias was evaluated by linear regression of the signed difference against the pairwise mean. No a priori endpoint-specific acceptance limits were defined for mean bias or limits of agreement. Accordingly, these analyses were intended to characterize measurement performance under the study conditions rather than to test formal equivalence or noninferiority against prespecified operational criteria. Because concordant zero BAC measurements could influence the overall agreement statistics, a secondary sensitivity analysis was performed among participants with nonzero reference BAC. For this subset, mean bias, 95% limits of agreement, mean absolute error, root mean square error, Lin’s concordance correlation coefficient, Deming-regression slope, and proportional bias were recalculated using the same analytical procedures as for the full cohort.

To evaluate whether participant characteristics influenced measurement performance, separate multivariable linear regression models were fitted for temperature, heart rate, systolic blood pressure, diastolic blood pressure, and blood alcohol concentration. Standardized absolute error was used as the dependent variable. Independent variables included age, sex, BMI, grouped Fitzpatrick skin type, eyeglass use, and the corresponding reference-measurement magnitude. Continuous predictors were standardized before model fitting.

Because Fitzpatrick skin types I (*n* = 4) and V (*n* = 5) were too sparsely represented to support reliable type-specific inference, skin type was analyzed using three grouped categories: I–II (*n* = 52), III (*n* = 190), and IV–V (*n* = 58). No separate inferential estimates were calculated for skin type I or V. Heteroskedasticity-consistent type 3 (HC3) standard errors were used to calculate 95% confidence intervals and *p* values. The Benjamini–Hochberg procedure was applied within each measurement endpoint to control the false-discovery rate across the model coefficients. All tests were two-sided, and an adjusted *p* value < 0.05 was considered statistically significant. Analyses were performed using R version 4.5.2.

### 2.10. Ethics Statement

The study was conducted in accordance with the Declaration of Helsinki and was reviewed and approved by the Public Institutional Review Board designated by the Ministry of Health and Welfare, Republic of Korea (approval no. P01-202607-01-094; 24 July 2026). All participants provided written informed consent before data collection. The screening outcomes generated by the Through-Pass system were used solely for research purposes and did not affect participants’ employment status or eligibility for duty.

## 3. Results

### 3.1. A Closed-Loop Architecture for Rapid Multimodal Screening

We first established the architecture of the Through-Pass system, a gate-based platform that integrates identity verification, contactless physiological measurements, local signal processing, and rule-based pre-duty screening within a single workflow (Figure 1). The system combined RFID-based identification with infrared temperature measurement, facial video acquisition for rPPG, and contactless breath-alcohol sensing.

Acquired signals were processed locally for preprocessing, quality assessment, and physiological parameter estimation before being transmitted to a server and database. A rule-based decision module then assigned one of three screening outcomes: FIT, CONDITIONAL, or UNFIT. The workflow incorporated two safeguards. Measurements that did not satisfy the predefined signal-quality criteria were rejected, and the participant was prompted to reposition and repeat the acquisition. In addition, CONDITIONAL and UNFIT outcomes were referred for review by authorized personnel rather than treated as final automated decisions. These outputs represent the implemented rule-based screening workflow and were not evaluated in the present study for criterion or predictive validity against independent duty-fitness or operational safety outcomes. The resulting architecture integrated multimodal measurement, quality control, screening classification, and human oversight within a single pre-duty process.

### 3.2. Quality-Gated Processing of Camera-Derived Physiological Signals

**Figure 2:**
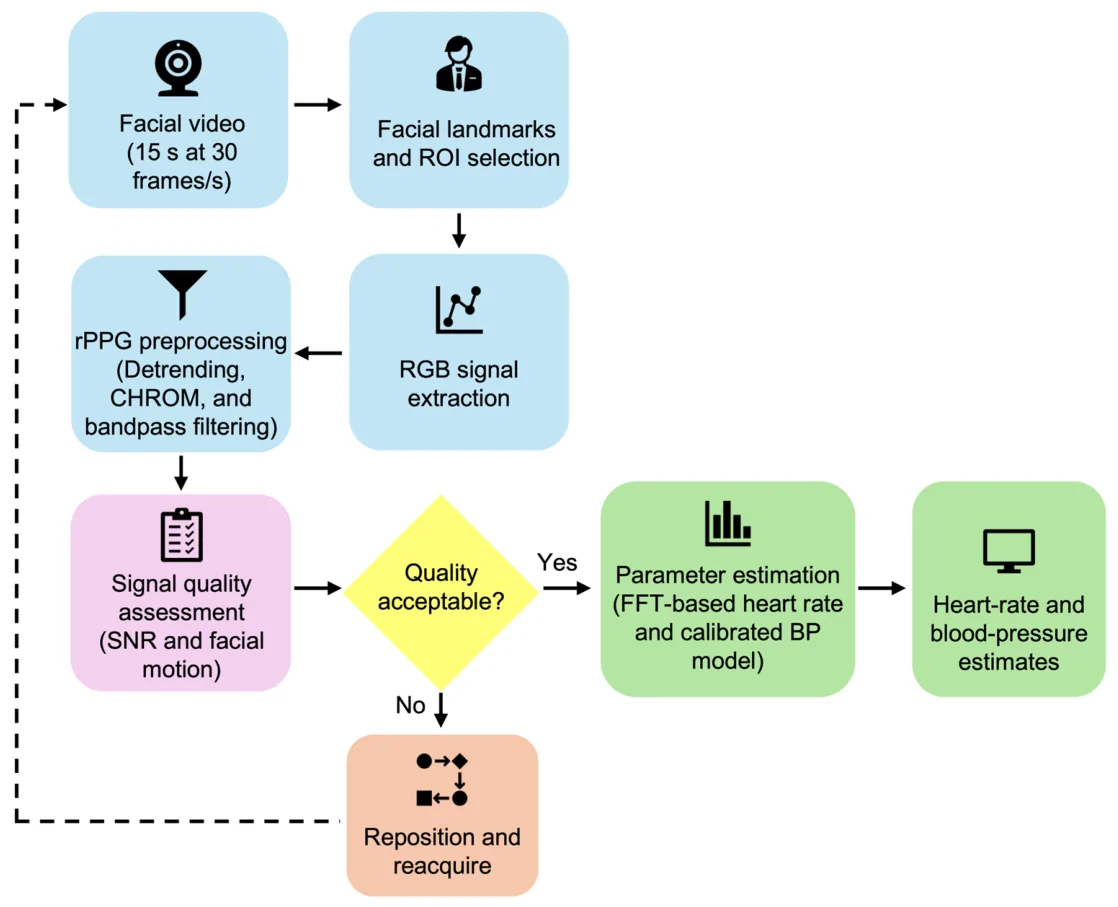
rPPG signal-processing and quality-control pipeline. Facial video was acquired for 15 s at 30 frames/s, followed by facial landmark detection, selection of facial ROIs, and extraction of RGB intensity signals. The signals were detrended and processed using the CHROM method and bandpass filtering to generate the rPPG waveform. Frequency-domain SNR was calculated as a signal-quality descriptor, while operational quality control used the ROI-stability, frame-stability, acquisition-validity, and rPPG-output criteria described in Section 2.3. Recordings that passed the required quality criteria were used for physiological parameter estimation, whereas failed recordings triggered participant repositioning and reacquisition. Heart rate was estimated using FFT-based frequency analysis, and systolic and diastolic blood pressures were estimated using the calibrated regression framework. Arrows indicate the direction of the processing workflow; colors and shapes are used to distinguish processing steps and do not represent additional quantitative variables.

We next characterized the camera-based processing workflow used to derive heart-rate and blood pressure estimates (Figure 2). During each acquisition, a 15 s facial video was recorded at 30 frames/s. Facial landmarks were detected, facial ROIs were selected from the face, and temporal RGB intensity signals were extracted. These signals were detrended and processed using the CHROM method and bandpass filtering to generate the rPPG waveform.

Before physiological values were reported, frequency-domain SNR was calculated as a signal-quality descriptor, while operational quality control followed the ROI-stability, frame-stability, acquisition-validity, and rPPG-output criteria described in Section 2.3. Recordings that failed the required quality criteria were rejected, and the participant was prompted to reposition and repeat the acquisition. Only accepted recordings proceeded to parameter estimation. Heart rate was derived using FFT-based frequency analysis, whereas systolic and diastolic blood pressures were estimated using the calibrated regression framework.

This quality-gated pipeline prevented unsuitable facial recordings from being automatically propagated into downstream estimation and screening classification. Together with the system-level workflow shown in Figure 1, it established an explicit link between signal acquisition, quality control, physiological estimation, and reacquisition when needed.

### 3.3. Physical Implementation and User Interface of the Through-Pass Prototype

**Figure 3:**
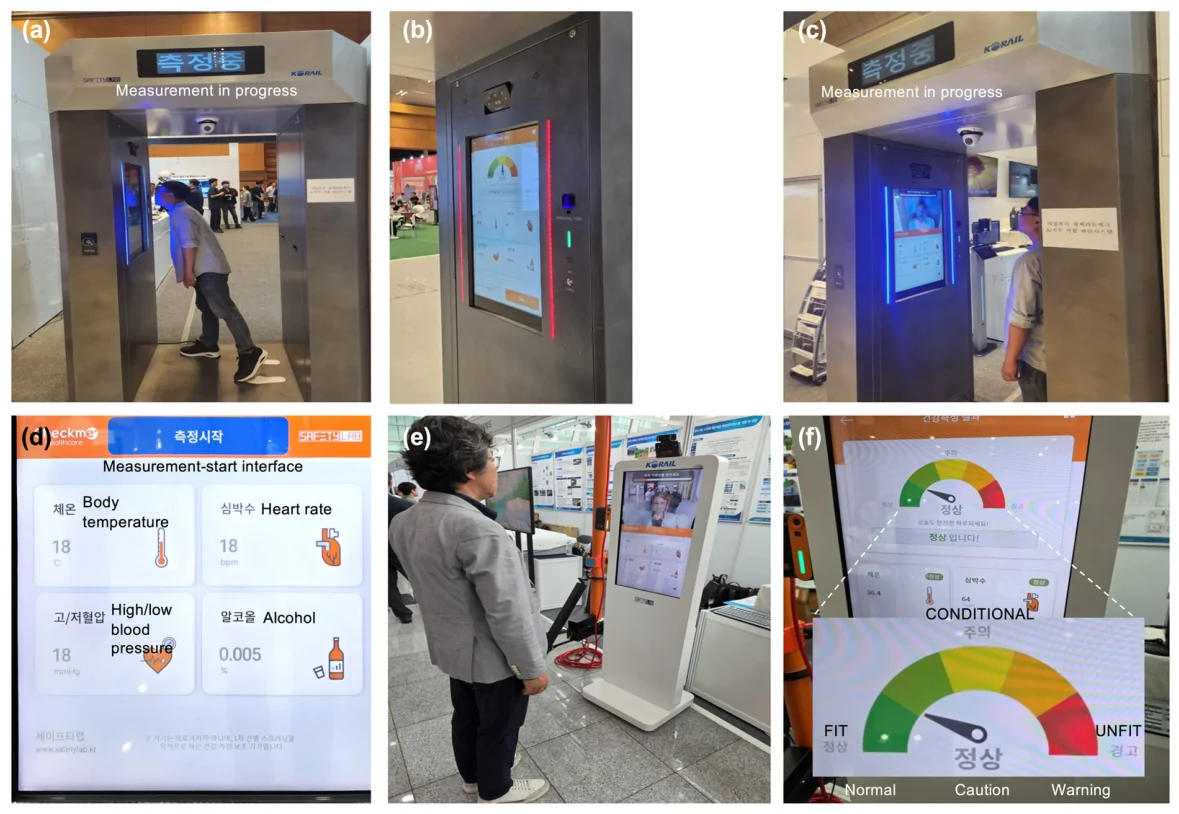
Physical implementation and user interface of the Through-Pass screening system. (**a**) Full view of the gate-based prototype during participant positioning and measurement. (**b**) Close-up of the integrated display, camera, radio-frequency identification reader, status indicators, and sensing area. (**c**) Three-quarter view showing the physical structure of the screening gate and the placement of the camera and user interface. (**d**) Measurement-start interface showing the temperature, heart rate, blood pressure, and breath alcohol fields; the displayed numerical values are demonstration placeholders rather than participant measurements. (**e**) Standalone demonstration unit used to evaluate camera-based signal acquisition and the user interface. (**f**) Example completed result screen displaying the measured parameters and overall screening status; the inset enlarges the FIT, CONDITIONAL, and UNFIT classification scale. The displayed FIT, CONDITIONAL, and UNFIT statuses illustrate the prototype rule-based interface and were not validated as predictors of duty fitness in the present study. English translations of the principal Korean interface labels are provided directly in the relevant panels.

The architecture and processing workflow described in Figure 1 and Figure 2 were implemented in a full-scale gate-based prototype (Figure 3). The camera, RFID reader, display, status indicators, and breath-alcohol sensor were integrated into a single passage structure, allowing participants to remain positioned within the gate while facial video and other sensor measurements were acquired during the acquisition period, which lasted approximately 15 s per attempt.

The user interface guided participants through measurement initiation, signal acquisition, and result display. Separate fields were provided for temperature, heart rate, blood pressure, and blood alcohol concentration. The numerical values shown in Figure 3d are demonstration placeholders rather than participant measurements. A standalone demonstration unit was also used to assess camera-based acquisition and interface operation outside of full gate configuration (Figure 3e).

After measurement, the interface displayed the estimated physiological values and an overall FIT, CONDITIONAL, or UNFIT screening status (Figure 3f). These displayed statuses illustrate the implemented screening and referral workflow and should not be interpreted as validated determinations of duty fitness. The physical implementation therefore demonstrated that participant identification, multimodal sensing, signal processing, quality control, and rule-based result presentation can be integrated into a single prototype. This prototype was subsequently used for the participant-level evaluation of measurement agreement; the validity of the screening statuses themselves was not evaluated in the present study.

### 3.4. Participant Characteristics

The evaluation cohort comprised 300 adults with a mean age of 38.3 ± 12.0 years (Table 1). The sex distribution was nearly balanced, with 144 male participants (48.0%) and 156 female participants (52.0%). Mean height and weight were 165.9 ± 8.6 cm and 63.9 ± 11.1 kg, respectively, and the mean BMI was 23.1 ± 3.1 kg/m^2^.

Based on BMI classification, 120 participants (40.0%) were in the normal-weight category, whereas 73 (24.3%) were overweight and 82 (27.3%) were obese. Twenty-five participants (8.3%) were underweight. Fitzpatrick skin type III was the most common category, accounting for 190 participants (63.3%), followed by type IV in 53 participants (17.7%) and type II in 48 participants (16.0%). Skin types I and V were uncommon, representing four (1.3%) and five (1.7%) participants, respectively. Eyeglasses were worn by 172 participants (57.3%).

Because skin types I and V were represented by only four and five participants, respectively, these categories were not analyzed separately in the inferential error models. Instead, Fitzpatrick skin type was grouped as I–II (*n* = 52), III (*n* = 190), and IV–V (*n* = 58). Accordingly, subsequent analyses assess differences among these grouped categories and do not provide type-specific estimates for skin type I or V.

### 3.5. Expanded Method Comparison Showed Low Bias and High Concordance with Reference Measurements

**Figure 4:**
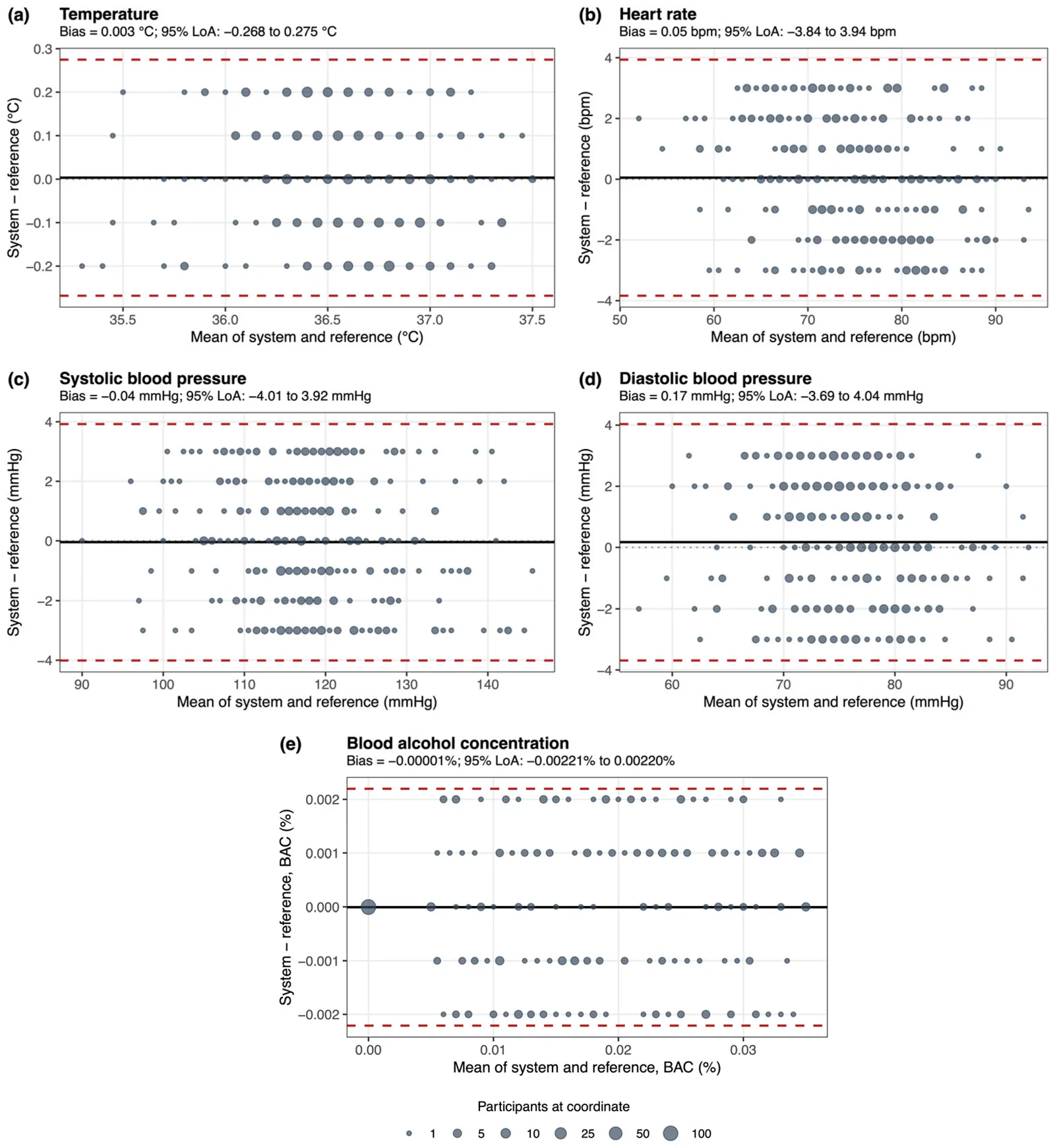
Bland–Altman analysis of agreement between the Through-Pass system and reference measurements in 300 participants. (**a**) Body temperature. (**b**) Heart rate. (**c**) Systolic blood pressure. (**d**) Diastolic blood pressure. (**e**) Blood alcohol concentration. For each participant, the difference was calculated as the Through-Pass measurement minus the corresponding reference measurement and plotted against the mean of the two measurements. Black solid lines indicate the mean bias, red dashed lines indicate the 95% limits of agreement, and gray dotted lines indicate zero difference. Point size represents the number of participants sharing the same plotting coordinate. Bias and 95% limits of agreement are reported above each panel.

Agreement between the Through-Pass system and the corresponding reference instruments was evaluated in 300 participants for body temperature, heart rate, systolic blood pressure, diastolic blood pressure, and blood alcohol concentration. SpO_2_ was not included in the agreement analyses because paired reference SpO_2_ measurements were not collected during the present study; consequently, no quantitative SpO_2_ performance results are reported. Individual-level differences are presented using Bland–Altman analysis in Figure 4, and complete agreement statistics are provided in Table S1. Across all five outcomes, mean system-minus-reference differences were close to zero. Lin’s concordance correlation coefficients ranged from 0.938 to 0.995, indicating high correspondence between the system and reference measurements.

For body temperature, the mean bias was 0.003 °C, with 95% limits of agreement from −0.268 to 0.275 °C (Figure 4a). Differences were distributed on both sides of zero across the observed temperature range. A small negative proportional trend was detected, indicating that the system-reference difference decreased slightly with increasing temperature (*p* = 0.032; Table S1).

Heart rate showed a mean bias of 0.05 bpm and 95% limits of agreement from −3.84 to 3.94 bpm (Figure 4b). Systolic blood pressure had a mean bias of −0.04 mmHg, with limits from −4.01 to 3.92 mmHg, whereas diastolic blood pressure had a mean bias of 0.17 mmHg, with limits from −3.69 to 4.04 mmHg (Figure 4c,d). Small negative proportional trends were detected for heart rate and systolic blood pressure (*p* < 0.001 and *p* = 0.048, respectively), whereas the corresponding trend for diastolic blood pressure was not statistically significant (*p* = 0.062). Despite these range-dependent trends, the Deming-regression slopes remained close to 1, ranging from 0.944 to 0.976 for the cardiovascular measurements. Sensitivity analyses using λ = 0.5 and λ = 2 produced similar Deming-regression slopes and did not alter the interpretation of agreement (Table S4). The horizontal bands in the heart rate and blood pressure panels reflect the integer-level resolution of the recorded values. Because measurements from the broader data-collection program contributed to population-level calibration and refinement of the blood pressure estimator, the systolic and diastolic blood-pressure agreement results should be interpreted as internal performance estimates rather than independent external validation results.

Blood alcohol concentration showed a mean bias of −0.00001% and 95% limits of agreement from −0.00221% to 0.00220% in the full cohort (Figure 4e). Of the 300 participants, 100 (33.3%) had a reference BAC of zero, whereas 200 (66.7%) had nonzero reference BAC values ranging from 0.005% to 0.035%. The concentration of observations at the origin in Figure 4e therefore represents overlapping zero-zero measurements rather than the majority of the cohort. Nonzero differences occurred in discrete increments, consistent with the measurement resolution of the alcohol-sensing system. No significant proportional bias was detected in the full cohort (*p* = 0.529).

To assess whether concordant zero measurements disproportionately influenced the overall BAC agreement statistics, a secondary sensitivity analysis was performed among the 200 participants with nonzero reference BAC. In this subset, the mean system-minus-reference bias remained −0.00001%, with 95% limits of agreement from −0.00271% to 0.00269%. The mean absolute error and root mean square error were 0.00117% and 0.00137%, respectively; Lin’s concordance correlation coefficient was 0.988. The Deming-regression slope was 1.010, and no significant proportional bias was detected (*p* = 0.364; Table S3). Thus, the overall BAC agreement was not attributable solely to concordant zero measurements. Nevertheless, the nonzero reference values covered a relatively limited range of 0.005–0.035%, and the present findings should not be extrapolated beyond the observed concentration range without further validation.

Overall, the expanded evaluation demonstrated mean biases close to zero and high concordance for temperature, heart rate, systolic and diastolic blood pressure, and blood alcohol concentration. Small range-dependent trends were observed for temperature, heart rate, and systolic blood pressure, emphasizing the importance of considering measurement behavior across the observed range rather than mean bias alone. The practical implications of these trends for threshold-based screening are considered in the Section 4. We next examined whether measurement-error magnitude varied with participant characteristics or the corresponding reference value.

### 3.6. Measurement Error Showed No Consistent Associations with Participant Characteristics

**Figure 5:**
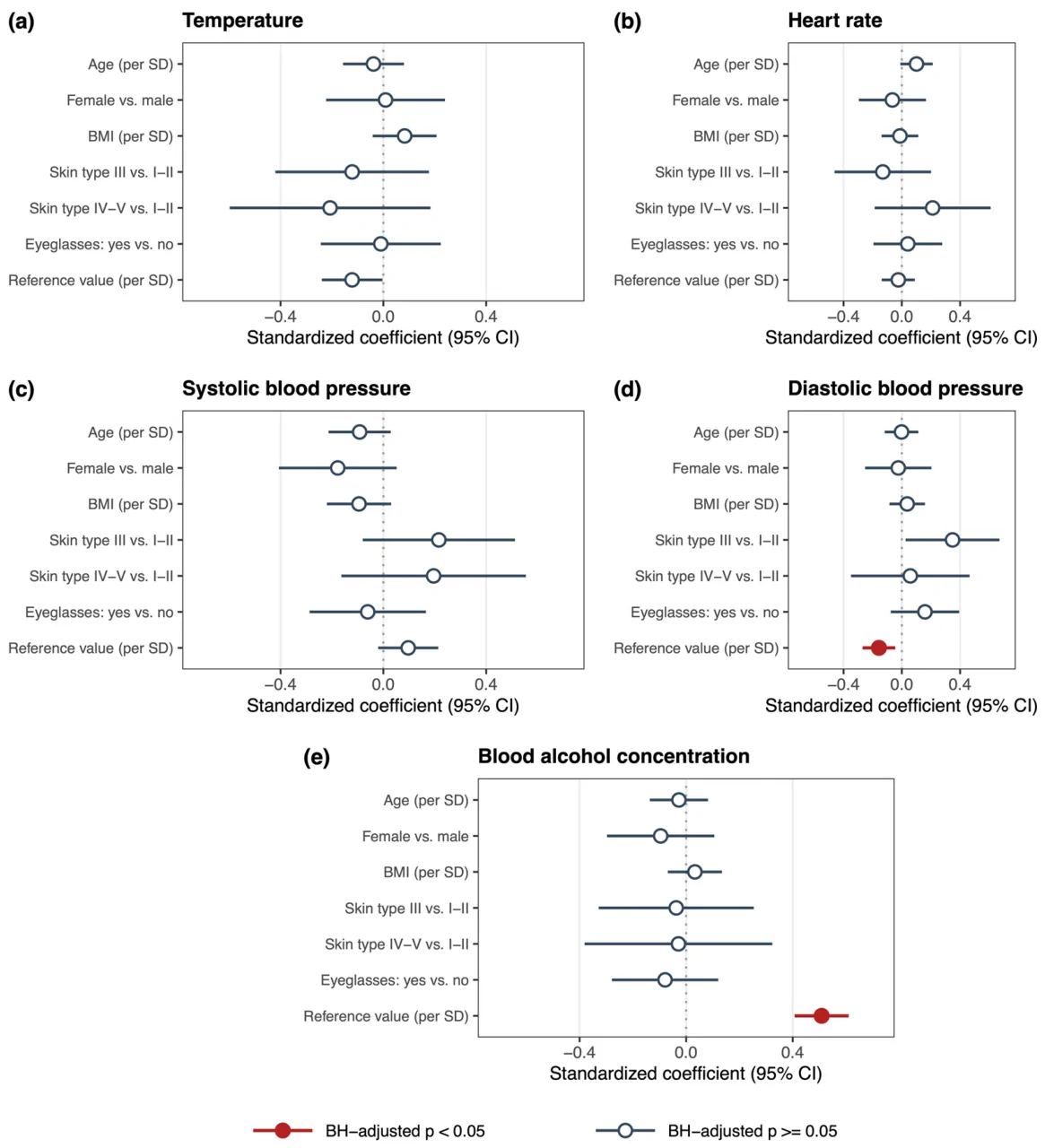
Adjusted associations between participant characteristics and absolute measurement error. Forest plots show results for (**a**) temperature, (**b**) heart rate, (**c**) systolic blood pressure, (**d**) diastolic blood pressure, and (**e**) blood alcohol concentration. Points represent standardized regression coefficients from endpoint-specific multivariable linear models, and horizontal lines indicate HC3 robust 95% confidence intervals. Positive coefficients indicate greater absolute error, whereas negative coefficients indicate lower absolute error. Continuous predictors were scaled per standard deviation; categorical comparisons were female versus male, grouped Fitzpatrick skin type III versus I–II and IV–V versus I–II, and eyeglass use versus no eyeglass use. The vertical dotted line indicates no association. Filled dark-red points denote Benjamini–Hochberg-adjusted *p* < 0.05 within the corresponding measurement endpoint, whereas open navy points denote adjusted *p* ≥ 0.05.

We next examined whether absolute measurement error varied according to age, sex, body mass index, grouped Fitzpatrick skin type, eyeglass use, or the corresponding reference-measurement magnitude. Separate multivariable models were fitted for temperature, heart rate, systolic blood pressure, diastolic blood pressure, and blood alcohol concentration. HC3 robust confidence intervals and Benjamini–Hochberg-adjusted *p* values were calculated within each endpoint (Figure 5; Table S2).

Age, sex, body mass index, grouped Fitzpatrick skin type, and eyeglass use were not significantly associated with standardized absolute error for any of the five measurement types after multiple-testing correction. For skin type, these comparisons were based on the grouped categories I–II, III, and IV–V and should not be interpreted as separate estimates for the sparsely represented individual type I or V. The corresponding confidence intervals included zero, indicating that the data did not provide evidence of consistent differences in measurement error across these participant characteristics. Several skin-type coefficients, particularly those for blood-pressure measurements, had comparatively large point estimates, but their confidence intervals were wide and crossed zero. These results should therefore be interpreted as imprecise estimates rather than evidence of a reproducible skin-type effect.

Two reference-value associations remained significant after Benjamini–Hochberg correction. For diastolic blood pressure, higher reference values were associated with lower standardized absolute error after adjustment for the other model covariates (Figure 5d). In contrast, higher reference blood alcohol concentrations were associated with greater absolute error (Figure 5e). Because the modeled outcome was absolute error, the BAC association indicates increasing disagreement in magnitude but does not indicate a consistent direction of overestimation or underestimation.

Overall, no consistent associations between the evaluated participant characteristics and measurement error were detected within the composition of this cohort. However, the grouped Fitzpatrick skin-type analysis does not establish equivalent measurement performance across individual skin types, particularly types I and V, which were sparsely represented. Significant reference-value terms indicated that error magnitude varied across the measurement range for diastolic blood pressure and BAC. For BAC, although the nonzero-reference sensitivity analysis showed that overall agreement was not driven solely by concordant zero measurements, the observed nonzero concentration range remained limited; performance across a broader range of BAC values therefore requires further evaluation.

## 4. Discussion

This study evaluated the system-level integration and participant-level measurement performance of a gate-based platform combining identity verification, contactless physiological measurement, signal-quality control, and rule-based pre-duty screening within a single workflow. In the expanded cohort of 300 participants, body temperature, heart rate, systolic and diastolic blood pressure, and blood alcohol concentration showed mean biases close to zero and relatively narrow limits of agreement with the corresponding reference measurements. Small proportional trends were detected for temperature, heart rate, and systolic blood pressure, but the Deming-regression slopes remained close to identity. These findings extend the initial prototype assessment by providing participant-level method-comparison data in a substantially larger cohort.

The findings are consistent with previous studies demonstrating that facial video can be used to derive heart rate and other cardiovascular information through remote photoplethysmography [13,14,16,25]. The principal contribution of the present study is, therefore, the integration of multiple sensing and decision components rather than the introduction of a new individual sensing method. Occupational monitoring has traditionally included wearable approaches for continuous physiological assessment during work [18,19], while more recent contactless transportation-monitoring systems have used camera-based physiological sensing during vehicle operation. These approaches primarily address continuous monitoring after duty has begun. In contrast, the Through-Pass platform is intended for brief stationary assessment before duty and integrates identity linkage, multimodal physiological measurement, explicit signal-quality gating, reacquisition, and screening/referral within a single workflow.

The blood-pressure results require an additional qualification. The estimator was developed using population-level rPPG and cuff-based reference measurements collected within the broader development program, and measurements from that program contributed to model calibration and refinement. The present systolic and diastolic blood-pressure agreement analysis therefore represents an internal performance evaluation rather than a fully independent external validation. Although no subject-specific calibration was required, independent evaluation in a cohort excluded from all stages of model development and calibration is necessary to establish the generalizability of the blood pressure estimator.

Age, sex, body mass index, grouped Fitzpatrick skin type, and eyeglass use were not significantly associated with absolute measurement error after multiple-testing correction. However, Fitzpatrick skin types I and V were represented by only four and five participants, respectively, and were therefore pooled with adjacent categories for the inferential analyses. The absence of significant differences among the grouped categories I–II, III, and IV–V should not be interpreted as evidence of equivalent measurement performance across individual Fitzpatrick skin types. Measurement magnitude was associated with error for two outcomes: absolute diastolic blood-pressure error decreased with increasing reference pressure, whereas absolute BAC error increased with reference concentration. Although the secondary analysis restricted to the 200 participants with nonzero reference BAC showed similarly high agreement, the observed nonzero BAC range was limited to 0.005–0.035%. Accordingly, the present findings should not be interpreted as demonstrating uniform BAC performance across all alcohol concentrations.

The small proportional trends observed for body temperature, heart rate, and systolic blood pressure also warrant consideration in the context of threshold-based screening. For each of these outcomes, the system-minus-reference difference became slightly more negative as measurement magnitude increased, indicating a tendency toward relatively greater underestimation at the higher end of the observed range. Although the Deming-regression slopes remained close to identity, mean bias alone does not fully characterize measurement performance across the range. In an operational screening setting, even a modest range-dependent shift could become relevant for measurements close to a decision threshold, where underestimation of an elevated value could potentially reduce the likelihood of triggering further review. Conversely, the present study did not define a priori acceptance limits or validate the screening thresholds against independent duty-fitness outcomes; therefore, the effect of these proportional trends on actual screening sensitivity, specificity, or misclassification cannot be determined from the present data. Future field validation should consequently evaluate agreement specifically around operationally relevant decision thresholds and across the full measurement ranges expected during routine use.

The present study did not prespecify endpoint-specific acceptance limits for bias or limits of agreement. Therefore, the observed near-zero mean biases, high concordance coefficients, and Deming-regression slopes close to identity should be interpreted as quantitative descriptions of measurement agreement rather than evidence that predefined operational acceptance criteria were met. This distinction is particularly important for threshold-based screening, where the practical significance of a given measurement difference depends on its proximity to the decision threshold and the consequences of misclassification. Future field-validation studies should therefore define endpoint-specific acceptance criteria prospectively and evaluate performance at operationally relevant thresholds before routine implementation.

Several limitations define the scope of the findings. First, evaluation was conducted under controlled indoor conditions rather than at an operational railway site. Consequently, the present results do not establish robustness to environmental and behavioral disturbances that may occur during routine use, including variable or nonuniform illumination, structural vibration, background activity, and unpredictable head or body motion. These factors are particularly relevant to camera-based rPPG because motion and illumination changes can affect facial-region tracking and pulse-signal extraction. Although the Through-Pass workflow incorporates signal-quality assessment and reacquisition when acquisition quality is insufficient, the present study did not quantify initial-acquisition success rate, reacquisition frequency, final acquisition failure rate, or total screening time. Accordingly, the approximately 15 s acquisition period represents the duration of a single attempt rather than guaranteed end-to-end screening throughput. The relatively short evaluation period also did not permit assessment of longitudinal hardware or sensor drift, repeated-use stability, or recalibration requirements. These operational factors should be evaluated during extended field deployment together with predefined calibration, maintenance, and quality-assurance procedures. Second, dedicated cross-interference testing of the MQ-3 alcohol sensor against potentially interfering volatile compounds was not performed; therefore, the present results establish agreement with the reference BAC measurements under the study conditions but do not establish the analytical selectivity of the sensor. In addition, although two-thirds of participants had nonzero reference BAC values, the observed nonzero range was limited to 0.005–0.035%, and performance at higher concentrations remains to be established. Third, SpO_2_ measurement capability was implemented in the prototype but was not quantitatively validated because paired reference SpO_2_ measurements were not collected during the present study. Accordingly, the present results provide no evidence regarding the accuracy or agreement of this functionality, which requires dedicated validation against an appropriate reference method before operational use. Fourth, the study evaluated agreement between the Through-Pass and reference measurements for individual physiological parameters rather than the validity of the FIT, CONDITIONAL, and UNFIT classifications or their association with operational safety outcomes. The decision module should therefore be regarded as a screening and referral mechanism rather than a diagnostic or independent employment-decision system. Further studies should evaluate measurement agreement and operational performance in actual railway pre-duty environments, assess BAC performance across broader concentration ranges and under potential cross-interference conditions, validate SpO_2_ measurement, independently evaluate the screening thresholds and classifications, and assess long-term system stability and recalibration needs.

## 5. Conclusions

This study evaluated the system-level integration and participant-level measurement performance of a Through-Pass gate platform, combining identity verification, contactless physiological sensing, signal-quality control, and rule-based pre-duty screening/referral. In 300 participants, measurements of body temperature, heart rate, systolic and diastolic blood pressure, and blood alcohol concentration showed mean biases close to zero and high concordance with the corresponding reference measurements under controlled indoor conditions. No consistent associations were detected between measurement error and age, sex, body mass index, grouped Fitzpatrick skin type, or eyeglass use, although error magnitude varied with reference diastolic blood pressure and blood alcohol concentration. Because no a priori operational acceptance limits were defined, these findings should be interpreted as participant-level evidence of measurement agreement rather than as a demonstration that predefined screening-performance criteria were met. The results support progression to field validation, including assessment of operational throughput, environmental robustness, longitudinal system stability, and independent validation of the FIT, CONDITIONAL, and UNFIT classifications before routine railway implementation.

## Figures and Tables

**Table 1:** Characteristics of the participants included in the expanded evaluation study.

Characteristic	Overall (*n* = 300) ^1^
Age, years	38.3 ± 12.0
Height, cm	165.9 ± 8.6
Weight, kg	63.9 ± 11.1
BMI, kg/m^2^	23.1 ± 3.1
Sex: Male	144 (48.0%)
Sex: Female	156 (52.0%)
BMI category: Underweight	25 (8.3%)
BMI category: Normal	120 (40.0%)
BMI category: Overweight	73 (24.3%)
BMI category: Obese	82 (27.3%)
Fitzpatrick skin type: I	4 (1.3%)
Fitzpatrick skin type: II	48 (16.0%)
Fitzpatrick skin type: III	190 (63.3%)
Fitzpatrick skin type: IV	53 (17.7%)
Fitzpatrick skin type: V	5 (1.7%)
Eyeglasses: No	128 (42.7%)
Eyeglasses: Yes	172 (57.3%)

^1^ Values are presented as mean ± standard deviation or number (%).

## Data Availability

The data supporting the findings of this study are available from the corresponding author upon reasonable request, subject to institutional and ethical approval. Participant-level data are not publicly available because they contain human-subject information and are subject to privacy and consent restrictions.

## Supplementary Materials

The following supporting information can be downloaded at: https://www.mdpi.com/article/10.3390/s26185805/s1. Table S1: Agreement statistics between the Through-Pass system and the corresponding reference measurements in 300 participants; Table S2: Adjusted associations between participant characteristics and standardized absolute measurement error; Table S3: Agreement statistics for blood alcohol concentration among participants with nonzero reference BAC values; Table S4: Sensitivity of Deming-regression slopes to the assumed measurement-error variance ratio.

## Abbreviations

The following abbreviations are used in this manuscript:

BAC	Blood alcohol concentration
BMI	Body mass index
CCC	Lin’s concordance correlation coefficient
CHROM	Chrominance-based method for rPPG signal extraction
CI	Confidence interval
FFT	Fast Fourier transform
HC3	Heteroskedasticity-consistent type 3 standard errors
LTE	Long-term evolution
MAE	Mean absolute error
RFID	Radio-frequency identification
RGB	Red, green, and blue
RMSE	Root mean square error
ROI	Region of interest
rPPG	Remote photoplethysmography
SNR	Signal-to-noise ratio
SpO_2_	Peripheral oxygen saturation
